# The effects of two gold-N-heterocyclic carbene (NHC) complexes in ovarian cancer cells: a redox proteomic study

**DOI:** 10.1007/s00280-022-04438-y

**Published:** 2022-05-11

**Authors:** Lara Massai, Luigi Messori, Andrea Carpentieri, Angela Amoresano, Chiara Melchiorre, Tania Fiaschi, Alessandra Modesti, Tania Gamberi, Francesca Magherini

**Affiliations:** 1 Department of Chemistry ‘Ugo Schiff’, University of Florence, via della Lastruccia 3-13, Sesto Fiorentino, 50019 Firenze, Italy; 2 Department of Chemical Sciences, University of Naples Federico II, Naples, Italy; 3 Department of Experimental and Clinical Biomedical Sciences, Mario Serio” University of Florence Viale G.B. Morgagni 50, 50134 Florence, Italy

**Keywords:** Carbene complexes, Ovarian cancer, Redox proteomic, Gold drugs

## Abstract

**Purpose:**

Ovarian cancer is the fifth leading cause of cancer-related deaths in women. Standard treatment consists of tumor debulking surgery followed by platinum and paclitaxel chemotherapy; yet, despite the initial response, about 70–75% of patients develop resistance to chemotherapy. Gold compounds represent a family of very promising anticancer drugs. Among them, we previously investigated the cytotoxic and pro-apoptotic properties of Au(NHC) and Au(NHC)_2_PF_6_, i.e., a monocarbene gold(I) complex and the corresponding bis(carbene) complex. Gold compounds are known to alter the redox state of cells interacting with free cysteine and selenocysteine residues of several proteins. Herein, a redox proteomic study has been carried out to elucidate the mechanisms of cytotoxicity in A2780 human ovarian cancer cells.

**Methods:**

A biotinylated iodoacetamide labeling method coupled with mass spectrometry was used to identify oxidation-sensitive protein cysteines.

**Results:**

Gold carbene complexes cause extensive oxidation of several cellular proteins; many affected proteins belong to two major functional classes: carbohydrate metabolism, and cytoskeleton organization/cell adhesion. Among the affected proteins, Glyceraldehyde-3-phosphate dehydrogenase inhibition was proved by enzymatic assays and by ESI–MS studies. We also found that Au(NHC)_2_PF_6_ inhibits mitochondrial respiration impairing complex I function. Concerning the oxidized cytoskeletal proteins, gold binding to the free cysteines of actin was demonstrated by ESI–MS analysis. Notably, both gold compounds affected cell migration and invasion.

**Conclusions:**

In this study, we deepened the mode of action of Au(NHC) and Au(NHC)_2_PF_6_, identifying common cellular targets but confirming their different influence on the mitochondrial function.

**Supplementary Information:**

The online version contains supplementary material available at 10.1007/s00280-022-04438-y.

## Introduction

Ovarian cancer is the second most lethal gynecological cancer and the seventh cause of cancer-related death in women around the world [Globocan 2020 https://gco.iarc.fr/]. Standard treatment consists of tumor debulking surgery followed by platinum and paclitaxel chemotherapy. Despite the initial response to this first-line treatment, about 70–75% of patients diagnosed with ovarian cancer develop recurrence of disease and drug resistance. Even though further chemotherapy opportunities are offered to these patients (such as the PARP inhibitors), survival rates have not substantially changed and five-year overall survival of all-stage ovarian cancer is still below 50%. Thus, the development of novel targeted therapies that retain activity against chemotherapy-resistant ovarian cancer is an unmet and urgent medical need.

Gold compounds form a variegate family of promising experimental agents for cancer treatment. Several gold(I) and gold(III) complexes manifested outstanding antiproliferative and cytotoxic properties in vitro against selected human cancer cell lines, and some of them were also tested successfully against in vivo cancer models [1–3]. The targets of antiproliferative gold compounds appear to be deeply distinct from those of clinically established platinum compounds but are still largely unexplored. Several studies indicated that gold compounds preferentially interact with proteins bearing free and solvent accessible thiol and selenol groups. Only a few direct protein targets for gold compounds have been discovered so far, such as the enzyme thioredoxin reductase (TrxR) [4, 5], the glycolytic enzyme hexokinase [6], the protein kinase PKCiota [7] and the IkB kinase (IKK) involved in the regulation of nuclear factor kB (NF-kB) [8]. In addition, many in vitro studies proved the strong interactions of gold(I) and gold(III) drugs with a series of model proteins, i.e., human serum albumin, carbonic anhydrase, superoxide dismutase, and hemoglobin [1, 9–11].

**Fig. 1:**
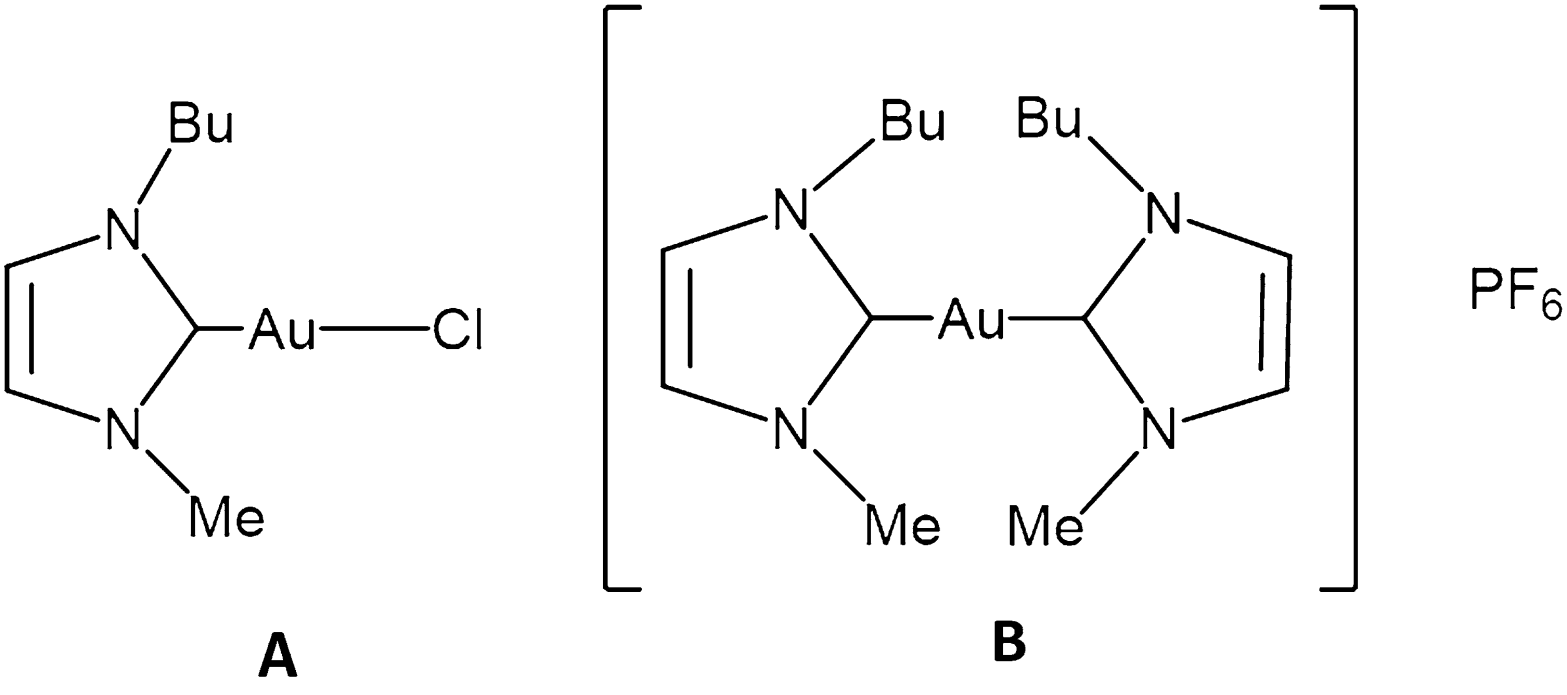
Structures of the NHC-gold(I) complexes: **A** chlorido (1-butyl-3-methyl-imidazole-2-ylidene) gold(I) **B** bis(1-butyl-3-methyl -imidazole-2-ylidene) gold(I)

Various gold-N-heterocyclic carbene (NHC) complexes have been prepared and characterized during the past few years as prospective anticancer agents and some of them turned out particularly effective and promising from the biological and pharmacological point of view [12–14]. In the last years we have synthesized, chemically characterized and biologically tested two gold(I) carbene complexes (Fig. 1) [15] that are structurally related. In fact, in both complexes, the gold(I) center linearly coordinates a 1-butyl-3-methyl-imidazole-2-ylidene ligand and, as second ligand, a chloride (complex **1**: Au(NHC)Cl, i.e., Au(NHC)) or another identical N-heterocyclic carbene (complex **2**: [Au(NHC)_2_]PF_6_, i.e., Au(NHC)_2_) The presence of a relatively labile ligand (chloride) in complex 1, makes the two compounds highly distinct from the chemical point of view, even in terms of the overall charge: the monocarbene complex is neutral, less stable and more reactive than its bis-carbene counterpart which is mono-cationic, highly stable and less reactive; however, Au(NHC)_2_ showed a greater biological activity than Au(NHC) in several experiments and this is probably due to its behavior as a delocalized lipophilic cation (DLC). In a previous study we demonstrated that both compounds produce strong antiproliferative and cytotoxic actions on ovarian cancer cells inducing apoptotic cell death [16]. Through implementation of a classical proteomic approach, we demonstrated that these gold carbenes affect importantly the amount of many proteins involved in protein synthesis, metabolism, cytoskeleton, and stress response. Notably, Au(NHC)_2_ was found to cause an evident upregulation of several glycolytic enzymes; Au(NHC)_2_ also triggered a metabolic shift towards glycolysis, probably due to an impairment of respiration. In fact, we observed a reduction of oxygen consumption and ATP amount, together with an increase of mitochondrial ROS production and a decrease of mitochondrial membrane potential. Both compounds inhibited TrxR activity as already shown for several other gold compounds.

In the present study, we go deeper in the analysis of the targets and the biological effects of these compounds. We hypothesized that Au(NHC) and Au(NHC)_2_ might cause an alteration of protein cysteine redox state both directly and indirectly. In fact, as we previously summarized, these compounds and similar gold complexes can directly interact with free cysteine and selenocysteine residues of several cellular proteins although the biological impact of this interaction has been investigated mostly for TrxR. On the other hand, the inhibition of TrxR can affect the cysteine redox state of other proteins causing what we have indicated as an indirect effect. In order to investigate these possibilities, we have performed a redox proteomics study aimed to find those proteins that are more prone to cysteine oxidation. We identified indeed a number of proteins with this propensity, mostly belonging to carbohydrate metabolism, cytoskeleton structure, protein folding and RNA interaction. Concerning carbohydrate metabolism, we found several enzymes that are more oxidized after gold carbene treatment; among these, we deeply investigated the effect of the treatment on GAPDH. We demonstrated that gold carbene compounds are able to inhibit the activity of this enzyme in treated cells and in cell extracts incubated with different amounts of gold complexes. In addition, to confirm a direct interaction, mass spectrometry of GAPDH-gold carbene compounds was performed and unambiguous evidence for adduct formation obtained. Moreover, we confirmed that only Au(NHC)_2_ impacts the mitochondrial function by inhibiting complex I activity. Subsequent functional studies have shown that both gold carbenes affect migration and invasion probably by interacting with actin. Again, this interaction was supported by ESI–MS analysis.

## Experimental procedures

### Cell lines, culture conditions and materials

A2780 human ovarian cancer were purchased from the European Collection of Authenticated Cell Cultures (ECACC, a part of Public Health England) (Lot No. 13J012, Sigma-Aldrich). Cells were maintained in RPMI-1640 medium supplemented with 10% of FCS and antibiotics at 37 °C in a 5% CO_2_ atmosphere and sub-cultured twice weekly.

Au(NHC) and Au(NHC)_2_ were synthesized in the MetMed Laboratories at the Department of Chemistry, University of Florence, following already established procedures [15]. LC–MS grade solvents and salts (water, DMSO and ammonium acetate) were purchased from Merck.

For drugs treatment, 4 × 10^4^/cm^2^ cells were seeded in p100 or in p60 plates and after 24 h were exposed to concentration of carbene complexes equal to their 72-h exposure IC50 values, i.e., 1.98 μM for Au(NHC) and 0.1 μM for Au(NHC)_2_ [16]. The drug incubations were stopped at 24 or 48 h depending on the specific experiment performed.

### Cysteine residues labeling.

A2780 cells were treated for 24 h with a concentration of the Au(NHC) or Au(NHC)_2_ equal to 72-hmexposure IC50 values (namely 1.98 and 0.1 µM respectively) and with an equal concentration of DMSO as control. After treatment, cells were washed with phosphate-buffered saline (PBS) and then lysed in RIPA buffer (50 mM Tris–HCl pH 7.0, 1% (v/v) NP-40, 150 mM NaCl, 2 mM ethylene glycol bis(2-aminoethyl ether)tetra-acetic acid, 100 mM NaF) containing a human protease inhibitor cocktail (Sigma-Aldrich) and 40 µM biotinylated iodoacetamide, BIAM (Molecular Probes). The cells were sonicated (15 s) and protein extracts were clarified by centrifugation at 8000*×g*, 4 °C for 15 min. Proteins were precipitated following a chloroform/methanol protocol and the protein pellets were resolved in a buffer containing 8 M urea, 4% (w/v) 3-cholamidopropyl dimethylammonium- 1-propane sulfonate (CHAPS), 65 mM dithioerythritol (DTE). The protein concentration was determined by the standard Bradford method (Bio-Rad Laboratories). Isoelectric focusing (IEF) was carried out on an IPGphor system (PROTEAN i12 system Bio-Rad Laboratories) using pH 3–10 gel strips of 11 cm. Strip were actively rehydrated at 50 V for 16 h in 200 μl of sample buffer, supplemented with 0.5% (v/v) carrier ampholyte (Bio-Rad Laboratories) and a trace of bromophenol blue, containing 200 μg and 80 μg of proteins for preparative gels and for gels to blot respectively. The IEF was performed at 20 °C under the following conditions: 250 V for 30 min, 10,000 V for 2 h in gradient, 10,000 V until a total of 43,000 Vh was reached, with a limiting current of 50 mA per strip. IPG strips were incubated for 15 min in equilibration buffer (50 mM Tris–Cl pH 8.8, 6 M urea, 30% glycerol, 2% SDS) with 2% DTT and then in equilibration buffer with 2,5% iodoacetamide for an additional 15 min. The equilibrated strips were placed on top of 8–16% precast polyacrylamide linear gradient gels (13.3 × 8.7 cm × 1.5 mm, BioRad) and embedded in 0.5% (w/v) heated low-melting agarose in SDS electrophoresis running buffer (25 m M Tris, 192 m M glycine, 0.1% (w/v) SDS, pH 8.3). SDS-PAGE was performed Criterion™ Vertical Electrophoresis Cell (Bio- Rad) at RT and at 200 V per gel, until the dye front reached the bottom of the gel. Preparative gels were stained with colloidal Coomassie blue silver. For western blot of 2D gels, proteins were transferred on PDVF membrane (Bio-Rad Laboratories). In all cases the blots were incubated for at least four hours with blocking buffer (PBS, 2% non-fat dry milk, 0.1%Tween-20). Biotinylated proteins were detected by incubating PVDF membrane with streptavidin-conjugated horseradish peroxidase (Biorad). The ECL system (GE Healthcare) was used for signal development. PVDF membranes were stained with Coomassie to check the transferring process and to confirm that the load was the same for each sample.

### Image analysis

For each condition, four gels from independent experiments were used for Western blot analysis. Gels and Western blot images were acquired with an Epson expression 1680 PRO scanner. Computer-aided 2D image analysis was carried out using the ImageMaster 2D Platinum 6.0 software (GE Healthcare). Relative spot volume (%*V* = *V* single spot/*V* total spots, where *V* is the integration of the optical density over the spot area) was used during analysis to reduce experimental error. The volume of carbonylated spots on oxyblots was normalized vs their respective spots visualized on Coomassie-stained gels. The efficiency and the reproducibility of the blotting was always checked by staining the PVDF membrane with brilliant blue Coomassie. Each Western blot image was matched with the image of Coomassie-stained gel of the same sample, to reveal the correspondence between BIAM signals and spots. Only the reproducible differences of BIAM signals were considered for protein identifications. A scheme of the workflow is shown in Fig. 1 of supplementary.

### Proteins identification by LC–MS/MS analysis

Trypsin, dithiothreitol, iodoacetamide and alfa-cyano-4-hydroxycinnamic acid were purchased from Sigma. Ammonium bicarbonate was from Fluka. Trifluoroacetic acid (TFA)-HPLC grade was from Carlo Erba. Acetonitrile and formic acid were purchase from Baker; bidistilled water (MilliQ) was provided by Millipore cartridge equipment. All other reagents and solvents were of the highest purity available from Carlo Erba, Rodano, Italy.

Selected spots were in situ hydrolyzed with trypsin and peptide mixtures thus obtained were directly analyzed by LTQ Orbitrap XL™ Hybrid Ion Trap-Orbitrap Mass Spectrometer (Thermo Fisher Scientific, Bremen, Germany). Chromatography on C-18 reverse phase capillary column 75 μm×10 cm (Thermo Fisher Scientific) was performed using a flow rate of 300 nl/min, with a gradient from eluent A (0.2% formic acid in 2% acetonitrile) to eluent B (0.2% formic acid in 95% acetonitrile). The following gradient conditions were used: *t* = 0 min, 5% solvent B; *t* = 10 min, 5% solvent B; *t* = 90 min, 50% solvent B; *t* = 100 min, 80% solvent B; *t* = 105 min, 100% solvent B; *t* = 115 min, 100% solvent B; *t* = 120 min; 5% solvent B. Peptides analysis was performed using data-dependent acquisition of one MS scan followed by CID fragmentation of the three most abundant ions. For the MS/MS experiment, we selected the three most abundant precursors and subjected them to sequential CID-MS/MS acquisition. For the MS scans, the scan range was set to 400–1800 m/z at a resolution of 60,000, and the automatic gain control (AGC) target was set to 1 × 106. For the MS/MS scans, the resolution was set to 15,000, the AGC target was set to 1 × 105, the precursor isolation width was 2 Da, and the maximum injection time was set to 500 ms. The CID normalized collision energy was 35%. Data were acquired by Xcalibur™ software (Thermo Fisher Scientific).

In-house Mascot software (version 2.4.0) was used as a search engine to identify proteins. The database used for protein identification was NCBInr 20150220 (61078976 sequences; 21793310741 residues), taxonomy: homo sapiens (human) (287718 sequences).

### MALDI-TOF mass spectrometry

To identify cysteines linked to BIAM reagent, peptide mixtures were analyzed by Positive Reflectron MALDI spectra were recorded on a 5800 MALDI-TOF/TOF (AB Sciex, Framingham, MA). The MALDI matrix was prepared by dissolving α-cyano-hydroxycinnamic powder in 70% acetonitrile and 30% citric acid 50 mM. Typically, 1 μl of matrix was applied to the metallic sample plate and 1 μl of analyte was added. The mixture thus obtained was then dried at room temperature. Mass calibration was performed using external peptide standards purchased from AB Sciex. MS spectra were acquired from 400 to 5000 m/z, for 10,000 laser shots total. Laser intensity remained fixed for all the analyses. Raw data were analyzed using the computer software provided by the manufacturers and are reported as monoisotopic masses. Spectra analysis was performed manually and sequence coverage was obtained by a peptide mass fingerprinting (PMF) approach [17].

### Electrospray ionization mass spectrometry experimental conditions

Sample preparation: stock solutions of GAPDH from rabbit muscle 10^− 4^ M (Merk G5262) and Actin from rabbit skeletal muscle 10^– 5^ M (Merck) were prepared dissolving the lyophilized protein in LC–MS grade water. Stock solutions 10^− 2^ M of the gold(I) carbene compounds were prepared by dissolving the samples in DMSO. For the experiments with GADPH aliquots of the stock solutions were mixed with aliquots of each gold carbene at protein-to-metal ratio 1:1 and diluted with ammonium acetate solution 2 × 10^− 3^ M (pH 6.8) to 10^− 5^ M final protein concentration. The mixtures were incubated at 37 °C up to 1 h. After the incubation time, all solutions were sampled and diluted to a final protein concentration of 5 × 10^− 7^ M using ammonium acetate solution 2 × 10^− 3^ M, pH 6.8 and adding 0.1% v/v of formic acid just before the infusion in the mass spectrometer.

For the experiments with actin, aliquots of the stock solutions were mixed with aliquots of each gold carbene at protein-to-metal ratio 1:1. The mixtures, at protein concentration of 10^– 5^ M were incubated at 37 °C up to 1 h. After the incubation time, all solutions were sampled and diluted to a final protein concentration of 5 × 10^− 6^ M LC–MS grade water and adding 0.2% v/v of formic acid just before the infusion in the mass spectrometer.

Instrumental parameters: the ESI mass study was performed using a TripleTOF 5600 + high-resolution mass spectrometer (AB Sciex, Framingham, MA, United States), equipped with a DuoSpray^®^ interface operating with an ESI probe. Respective ESI mass spectra were acquired through direct infusion at 7 μL/min of flow rate. The general ESI source parameters optimized for the proteins analysis were as follows(GAPDH) positive polarity, ion spray voltage floating 5500 V, temperature 25 °C, ion source Gas 1 (GS1) 20 L/min; ion source Gas 2 (GS2) 0; curtain gas (CUR) 15 L/min, collision energy (CE) 10 V; declustering potential (DP) 200 V, acquisition range 1400–4500 m/z; (ACTIN) positive polarity, ion spray voltage floating 5500 V, temperature 25 °C, ion source Gas 1 (GS1) 20 L/min; ion source Gas 2 (GS2) 0; curtain gas (CUR) 15 L/min, collision energy (CE) 10 V; declustering potential (DP) 100 V, acquisition range 765–2000 m/z For acquisition, Analyst TF software 1.7.1 (Sciex) was used, and deconvoluted spectra were obtained by using the Bio Tool Kit micro-application v.2.2 embedded in PeakViewTM software v.2.2 (Sciex).

### Glyceraldehyde-3-phosphate dehydrogenase enzymatic activity inhibition assay in cell extracts

A2780 cells were seeded at 4 × 10^4^ cells/cm^2^ in RPMI-1640 complete medium for 24 h. Cells were washed with phosphate-buffered saline solution and lysed in the GAPDH assay buffer (50 mM triethanolamine, pH 8.9, 0.2 mM EDTA, and 50 mM K2PO4) containing human protease inhibitor cocktail (Sigma). The protein contents were determined by a Bradford Reagent (Biorad) and 10 µg of protein were pre-incubated for 5 min with different concentrations of the metal complexes ranging from 100 and 0.5 µM. Afterwards the enzyme activity was initiated by the addition of 2 mM glyceraldehyde-3-phosphate and GAPDH activity was measured for 40 s at 25 °C via the change in absorbance at 340 nm using UV 1800 spectrophotometer (Scimadzu). The EC50 values were determined in triplicate experiments using dose-response fitting equation of GraphPad Prism software. The EC50 value is defined as the complex concentration causing a decrease of the enzyme activity to 50%.

### Cell-based glyceraldehyde-3-phosphate dehydrogenase enzymatic activity inhibition

Protein extracts were obtained from A2780 cells untreated (control) or treated with compounds for 24-h exposure at their respective 72-h IC50 values**.** At the end of incubation, cells were washed with phosphate-buffered saline solution and lysed GAPDH assay buffer. The protein contents were determined by a Bradford Reagent (Biorad). 10 µg of total proteins were used for the enzymatic assay. Three technical replicates were performed for three independent biological experiment.

### Cell migration and invasion

Cell migration was evaluated by wound-healing assay and by migration on Boyden chamber. For wound-healing assay, A2780 cells were seeded onto p60 plates and allowed to growth at confluence. The cells were then treated with Au(NHC) and Au(NHC)_2_ in culture medium in absence of fetal bovine serum for 24 h afterword the monolayer was scratched using micropipette tip to create a gap with no cells. The wound was photographed immediately after the scratch and after 16 h with the aid of an inverted microscope. At least twenty images for each condition were taken in three independent biological experiment. The reduction of the wound area was measured using the Image J software.

For migration on Boyden chambers, 100.000 cells were seeded onto Transwell Permeable Support inserts with 8 lm micro-porous membrane (Greiner bio-one) in culture medium without fetal bovine serum and containing gold compounds. After 24 h, complete medium was added to the lower compartment of the plate. After 16 h, the cells on the upper surface of the membrane were wiped out using a cotton swab. The cells that migrated to the lower surface were fixed and stained with crystal violet. The stained cells were photographed and counted under a microscope. Invasion assay was performed with the same method but Transwell inserts were pre-coated with 50 µl of Matrigel (1.2 mg/ml, in serum-free RPMI-1640 medium). The experiment was performed in triplicate and at least ten randomly selected fields were counted for each chamber.

### Measurement of mitochondrial respiration activity in permeabilized cells

Intact cells were suspended in 0.5 ml of reaction buffer containing 0.25 M sucrose, 20 mM Tris–HCl, 4 mM MgSO_4_, 0.5 mM EDTA, 10 mM KH_2_PO_4_ (pH 7.4). 1 ml of cell suspension (10^6^ cells) was permeabilized by the addition of 45 μg of digitonin for 1 min. 5 mM glutamate/2.5 mM malate and 10 mM succinate were used, respectively, to induce complex I- and complex II-guided respiration to record State 4 (without ADP; non-phosphorylating mitochondria) rates. The addition of 0.1 mM ADP was then performed to record State 3 (with ADP; phosphorylating mitochondria) rates. The RCR (respiratory control ratio) was obtained by dividing the rate of oxygen consumption in State 3 (expressed as nmol of oxygen/min, obtained with a respiratory substrate and ADP) by that of State 4 (in the presence of a substrate without ADP).

### Western blot analysis

A2780 cells were treated for 48 h with carbene complexes at a concentration corresponding to their 72-h exposure IC50 dose. Cells were lysed in RIPA buffer containing a human protease inhibitor cocktail and 20 μg of proteins were separated by 4–20% precast SDS-PAGE (Bio-Rad Laboratories) and transferred to PVDF membranes (Bio-Rad Laboratories). Primary antibody (Total OXPHOS Human WB Antibody Cocktail (Abcam, ab110411) were diluted 1:1000 in 2% milk and incubated overnight at 4 °C. This antibody cocktail contains 5 different antibodies, each against protein of a specific complexes easily distinguishable by MW (antibody against subunit NDUFB8 for Complex I). After incubation with horseradish peroxidase (HRP)-conjugated anti-mouse IgG (1:5000) (Santa Cruz Laboratories), immune complexes were detected with the enhanced chemiluminescence (ECL) detection system (GE Healthcare) and images were acquired with Amersham Imager 600 instrument. For quantification, the blot was subjected to densitometric analysis using ImageJ2 program [18]. The intensity of the immunostained bands was normalized with the total protein intensities measured by Coomassie brilliant blue R-250 from the same PVDF membrane blot.

### Statistical analysis

Statistical analysis was performed by one-way ANOVA test followed by Tuckey’s multiple comparisons test using GraphPad Prism 6. A *p* value ≤ 0.05 was considered statistically significant. Results were reported as mean ± SD.

## Results

### Identification of oxidation-sensitive cysteines in protein from cells treated with Au(NHC) and Au(NHC)_2_

**Fig. 2:**
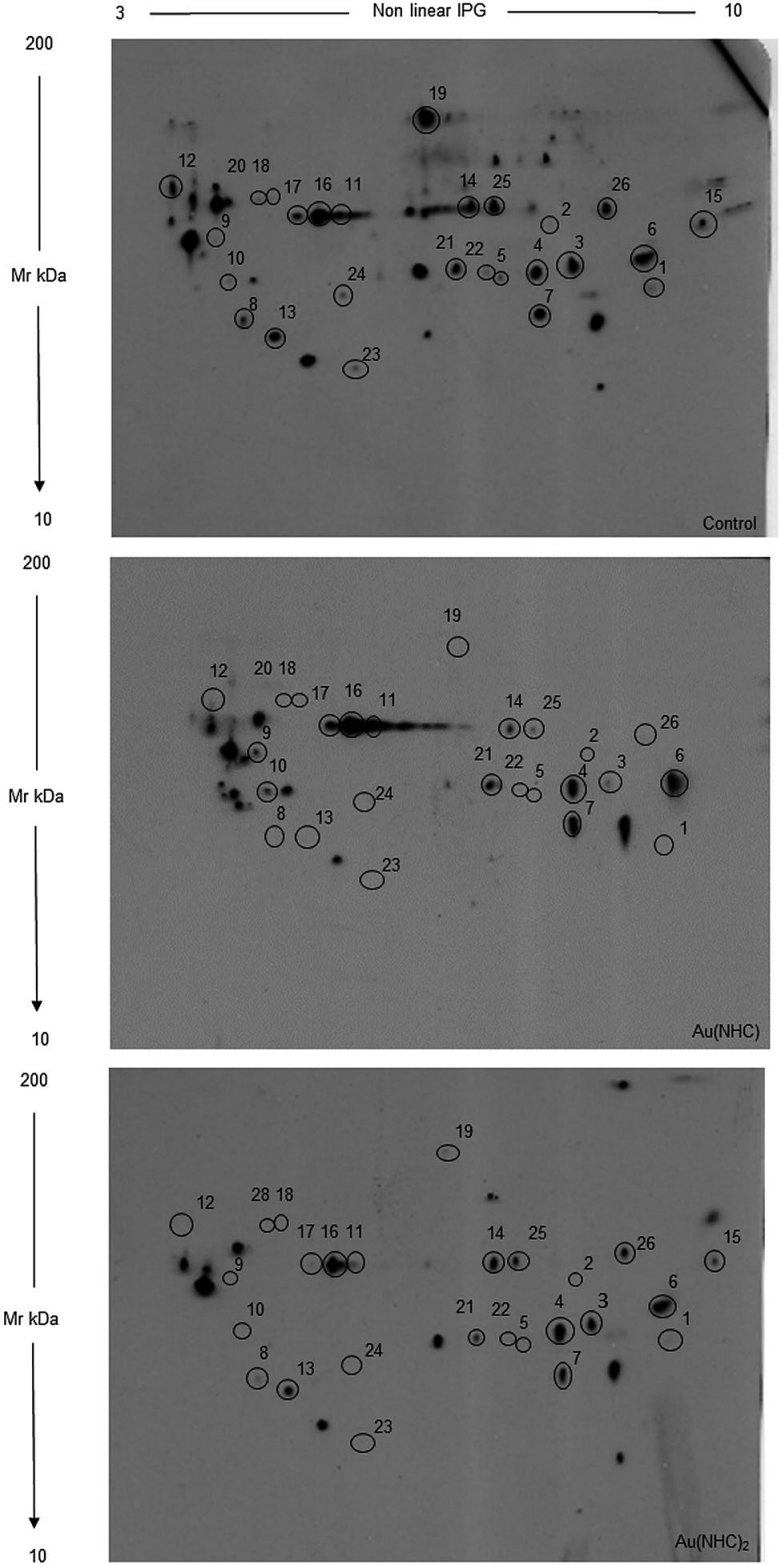
Redox status of cysteine residues in control and treated cells. Cells were harvested after 24 h of treatment and lysates were incubated with BIAM 40 µM. Proteins (100 μg) were separated by 2D-GE, transferred on PDVF membranes and incubated with streptavidin-HRP to detect biotinylated proteins. Representative images were reported. Black circles and numbers indicate differentially biotinylated spots, as reported in Table 1

Several studies have demonstrated that gold complexes directly interact with free cysteine and selenocysteine residues causing the formation of stable adducts [9–11]. Furthermore, we have previously shown that, in A2780 ovarian cancer cells, Au(NHC) and Au(NHC)_2,_ cause a remarkable inhibition of TrxR activity and Au(NHC)_2_ induces a net increase of mitochondrial ROS. The TrxR inhibition, the ROS increase and the direct interaction of gold compounds with cysteine residues can produce their oxidation decreasing the concentration of reduced SH groups. To evaluate this process, redox proteomics has been performed using the BIAM reagent that selectively links reduced cysteines. Protein lysates from control and gold treated cells (for 24 h with 72-h IC50) were separated by 2D-GE and the BIAM-labeled proteins were revealed with streptavidin-conjugated horseradish by Western blot (Fig. 2). ImageMaster 2D Platinum 6.0 software was used to identify proteins differentially labeled with the BIAM reagent. After spot detection, in lysates from control cells, 110 spots corresponding to BIAM labeled proteins were always detected and quantified in four independent experiments. Among these, 85 and 84 spots from Au(NHC) and Au(NHC)_2_ treated cells presented an intensity level not statistically different from the control, thus indicating that the cysteine residues of the corresponding proteins do not present a quantitative alteration of their reduced form upon treatment. 24 and 26 spots, respectively, from Au(NHC)- and Au(NHC)_2_-treated cells showed a clear reduction of intensity and, bona fide corresponded to proteins that underwent cysteine oxidation during treatment (the detection of increased amounts of biotinylated protein corresponds to less cysteine modifications and vice versa). For these spots, the fold change respect to control was calculated for each replicate. Blue Coomassie-stained gels were used for normalization of the data to exclude differences due to variations in the protein amount. Only the spots showing a fold change variation in all the four replicates were taken in account. For the majority of the spots fold change values changed largely between replicates although always detected, thus we have indicated the range of variation rather than the actual value accompanied by the standard deviation. Spots corresponding to differentially biotinylated proteins were picked from preparative Coomassie-stained gels (Fig. 2 of supplementary) and analyzed by MS. Table 1 reports the identity of these spots together with variations of intensity of western blot signal. The BIAM-linked peptide, if identified among peptides detected with MS analysis, has been reported in the Table. More detailed, MS data were reported in Table 1 of supplementary. In some spots, two or more different proteins with similar molecular weight and isoelectric point were identified; in this case, all proteins were reported. Most of the identified proteins belong to two major categories: carbohydrate metabolism and cytoskeleton organization, whilst a few of them belong to the protein folding and RNA-binding classes.

**Table 1:** Differentially BIAM-linked protein identified by mass spectrometry analysis

Spot *n*°^a^	Protein name	Accession number (UniProtKB)^b^	Biotinylated peptide	Au(NHC) vs control^c^	Au(NHC)_2_ vs control^c^
Carbohydrate metabolism					
1	Glyceraldehyde-3-phosphate dehydrogenase	P04406		≥ − 10	N.D^$^
2	Alpha-enolase	P06733		N.D	N.D
3	Alpha-enolase	P06733		≥ − 10	− 2 ≤ − 5
5	Alpha-enolase	P06733		N.D	N.D
6^*^	Fructose-bisphosphate aldolase A	P04075		− 2 ≤ − 5	− 2 ≤ − 5
6^*^	Fructose-bisphosphate aldolase C	P09972	CIGGVIFFHETLYQKDDNGVPFVR	− 2 ≤ − 5	− 2 ≤ − 5
6^*^	Phosphoglycerate kinase 1	P00558	ACANPAAGSVILLENLRFHVEEEGKGK	− 2 ≤ − 5	− 2 ≤ − 5
7^*^	Malate dehydrogenase, cytoplasmic	P40925	AICDHVR	− 2 ≤ − 5	− 2 ≤ − 5
8	l-lactate dehydrogenase B chain	P07195		N.D	− 2 ≤ − 5
Cytoskeleton organization and cell adhesion					
7^*^	Calponin-2	Q99439	CASQSGMTAYGTR	− 2 ≤ − 5	− 2 ≤ − 5
11	Actin, cytoplasmic 1	P60709	CDVDIRK	− 2 ≤ − 5	− 2 ≤ − 5
12	Tubulin beta chain	P07437	MREIVHIQAGQCGNQIGAK NMMAACDPRHGRYLTVAAVFR	− 2 ≤ − 5	N.D
13	F-actin-capping protein subunit alpha-1	P52907		≥ − 10	− 2 ≤ − 5
15	Serpin H1	P50454		≥ − 10	− 2 ≤ − 5
Protein folding					
9	Hsc70-interacting protein	P50503	AFVKMCK	− 2 ≤ − 5	− 2 ≤ − 5
16	Protein disulfide-isomerase A3	P30101		− 2 ≤ − 5	− 2 ≤ − 5
17	Protein disulfide-isomerase A3	P30101		− 2 ≤ − 5	− 2 ≤ − 5
18^*^	T-complex protein 1 subunit epsilon [Homo sapiens]	P48643		N.D	N.D
18^*^	T-complex protein 1 subunit theta [Homo sapiens]	P50990		N.D	N.D
19^*^	Heat shock protein HSP 90-beta	P08238	AKFENLCK KCLELFSELAEDKENYK CLELFSELAEDKENYKK	N.D	≥ − 10
19^*^	Heat shock protein HSP 90-alpha	P07900	DYCTR DYCTRMKENQK	N.D	≥ − 10
20	T-complex protein 1 subunit epsilon [Homo sapiens]	P48643		No variation	ND
RNA splicing and RNA binding					
4	Poly(rC)-binding protein 1	Q15366	GGCKIKEIR	− 2 ≤ − 5	− 2 ≤ − 5
21	Poly(rC)-binding protein 2	Q15366		− 2 ≤ − 5	− 2 ≤ − 5
22^*^	RNA-binding protein 4	Q9BWF3	SLFEQYGKVLECDIIK	N.D	N.D
Others					
10	Glutaredoxin-3 [Homo sapiens]	O76003		No variation	− 5 ≤ − 10
14	d-3-phosphoglycerate dehydrogenase	O43175	VVNCAR	≥ − 10	− 2 ≤ − 5
19^*^	Programmed cell death 6-interacting protein		CSDIVFAR	N.D	≥ − 10
23	Peflin	Q9UBV8		N.D	N.D
22^*^	Mitotic checkpoint protein BUB3	O43684	YQTRCIR	N.D	N.D
24	Band 3 anion transport protein	P02730		≥ − 10	≥ − 10
25	Inosine-5'-monophosphate dehydrogenase 2	P12268	HGFCGIPITDTGR	≥ − 10	− 2 ≤ − 5
26^*^	ATP synthase subunit alpha, mitochondrial	P25705		≥ − 10	− 5 ≤ − 10
26^*^	Glutathione reductase, mitochondrial	P00390		≥ − 10	− 5 ≤ − 10

Spots have been classified based on the Gene Ontology (GO) terms related to their major biological functions using UniProtKB database (http://www.uniprot.org/). Several proteins were associated with more than one function, and in such case, one category was chosen arbitrarily. The spots in which biotinylated peptides were identified are in bold and the peptide is reported

Detailed MS data were reported in Table 1 of supplementary

^a^Spot numbers match those reported in the representative 2-DE images shown in Fig. 2

^b^Accession number in Swiss-Prot/UniProtKB (http://www.uniprot.org/)

^c^Range of fold change, refer to the text for more details

^*^Indicates spots in which two or more different proteins were identified by MS

^§^N.D: indicates that the biotinylated spot has not been detected in treated cells vs control cells

### Interaction of gold carbenes with GAPDH and actin proved by ESI–MS analysis

**Fig. 3:**
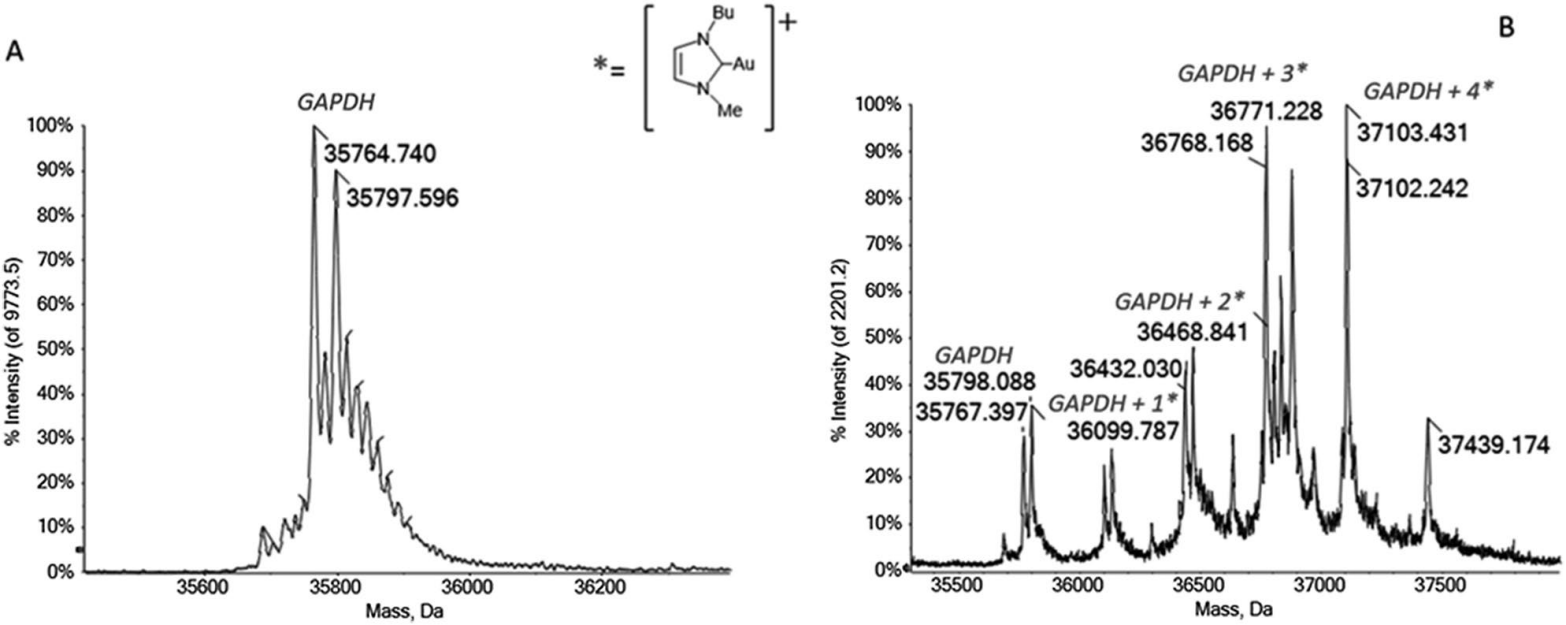
**A** Deconvoluted mass spectra of GAPDH 5 × 10^− 7^ M in 2 mM of ammonium acetate solution at pH 6.8. **B** Au(NHC) incubated with GAPDH, at 37 °C for 1 h in 1:1 protein-to-gold ratio; 0.1% v/v of formic acid was added just before infusion

Among the proteins that resulted oxidized upon Au(NHC) or Au(NHC)_2_ treatment, we found the enzyme glyceraldehyde-3-phosphate dehydrogenase (GAPDH) and actin, two proteins which play a pivotal role, respectively, in cell energetic metabolism and in cytoskeleton rearrangement. To better understand the mechanism beyond the oxidation of these proteins, we analyzed the interactions of GADPH and actin with the two gold carbenes; a few binding studies were conducted according to a well-defined experimental protocol that was developed in our laboratory in recent years and is now documented by several papers [10, 11, 15]. The results obtained by reacting GAPDH from rabbit muscle with the two gold carbene compounds in a 1:1 protein-to-metal ratio, are reported in comparison to the ESI mass spectrum of GAPDH alone (Fig. 3). The deconvoluted mass spectrum of the protein, Fig. 3A, is characterized by the main signal at 35764 Da assigned to the native protein and another peak at 35797 Da most likely due to the Cys150 sulfhydration. Upon addition of 1 equivalent of Au(NHC) a few intense peaks are detected, indicating extensive protein metalation both after 5 min and 1 h. The binding stoichiometry of metal fragment/GAPDH is up to 4:1, the metal fragments that bind the protein correspond to 1-butyl-3-methylimidazole-2-ylidene-gold(I).

Given that gold(I) compounds are highly thiophilic, we might assume that the cysteine residues could be a preferential binding site for the gold(I) carbene. Indeed, Glyceraldehyde-3-phosphate dehydrogenase possesses three free cysteine residues (Cys150, Cys 245 and Cys 282, referred to GAPDH from rabbit), which might explain the elevated binding stoichiometry. Furthermore, the adduct with 1:4 stoichiometry could be explained given the fact that other additional amino acid residues, i.e., His, Gln and Lys, showed an appreciable affinity for gold compounds, as demonstrated by several papers [19]. For the bis-carbene gold complexes, generally, a lower reactivity respect to the corresponding monocarbene gold complexes is generally observed by ESI–MS analysis; in this case, when studying the interaction of GADPH with Au(NHC)_2_, no signals of possible adducts were detected.

**Fig. 4:**
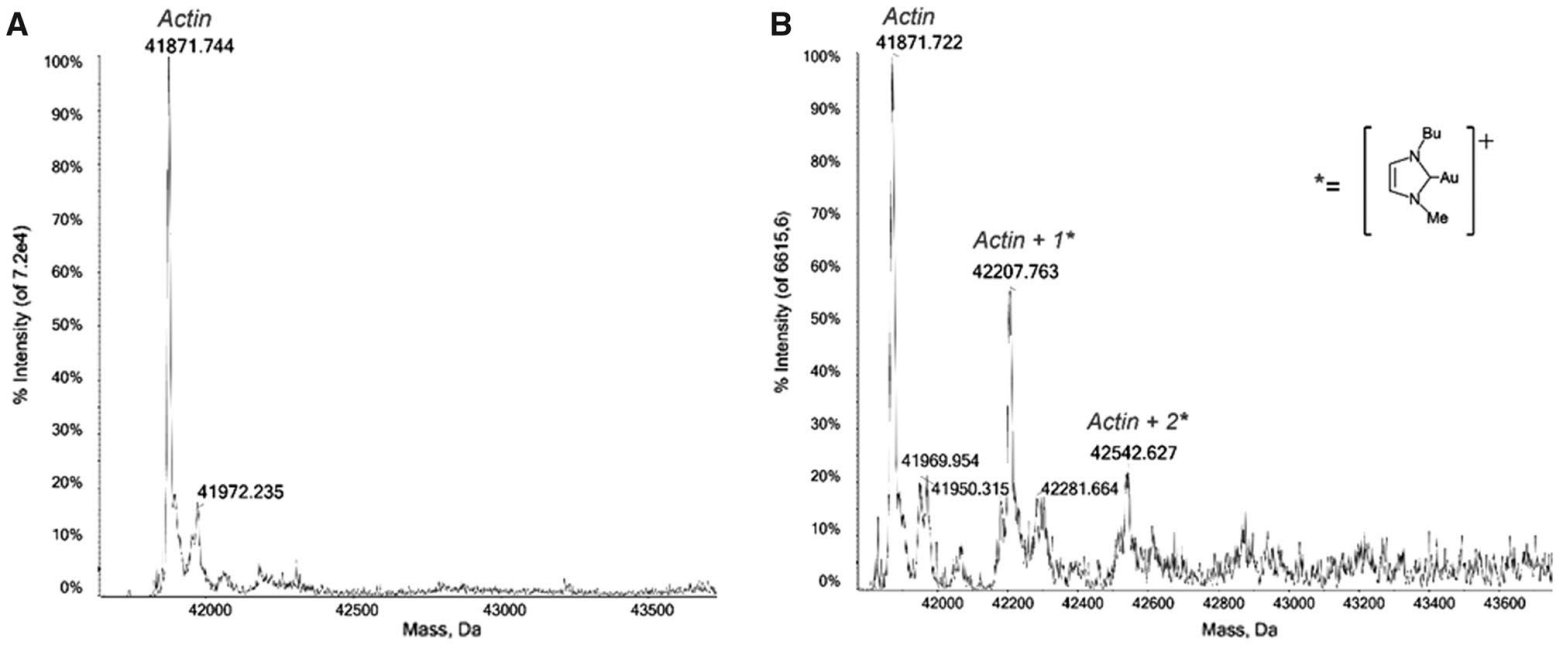
**A** Deconvoluted mass spectra of Actin 5 × 10^− 6^ M in LC–MS grade water. **B** Au(NHC) incubated with Actin, at 37 °C for 1 h in 1:1 protein-to-gold ratio; 0.2% v/v of formic acid was added just before infusion

Concerning the interaction between the gold carbene compounds and actin, in Fig. 4 the mass spectrum of actin from rabbit muscle with Au(NHC)Cl after 1 h of incubation is reported: beyond the signal of the protein alone, other signals at greater masses emerge. At 42207 Da is the signal of the mono adduct of the protein with the aforementioned [Au(NHC)]^+^ containing fragment, also the bis adduct signal is detected at 42542 Da.

Au(NHC) proved to be a highly reactive compound towards actin: it loses the labile ligand, the chloride, and binds to the protein as a cationic fragment. The protein reacts giving rise to a series of adducts bearing one or two copies of the gold cationic fragment. At variance, as observed in the case of GAPDH, Au(NHC)_2_ does not react over the 24 h of incubation with actin and no adducts are detected, pointing out a complete inertia of Au(NHC)_2_ to react with the selected proteins.

### Inhibition of GAPDH activity

#### Inhibition in cells pre-treated with gold carbene compounds

**Fig. 5:**
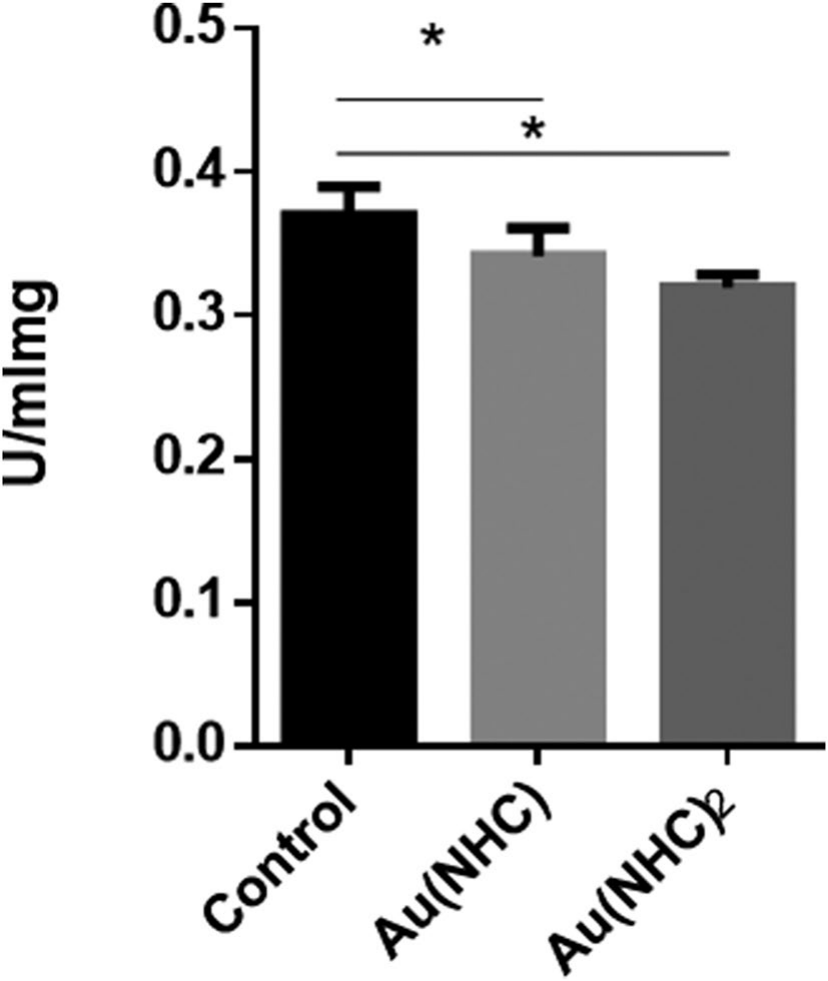
Au(NHC) and Au(NHC)_2_ inhibited GAPDH activity. A2780 cells treated for 24 h with Au(NHC) and Au(NHC)2 at their respective IC50 72-h concentrations. GAPDH was assayed on cell extracts. Data are means ± standard deviations of three independent experiment

Redox proteomic analysis showed that GAPDH enzyme resulted oxidized after Au(NHC) and Au(NHC)_2_ treatment; furthermore, Au(NHC) directly reacts with the enzyme, as we described in the previous paragraph. To verify if this oxidation/interaction could affect the enzyme activity, we treated A2780 cells for 24 h with compounds at their IC50 at 72 h. GAPDH activity was measured in cell extract as described in Experimental Procedure. Three biological independent experiments were performed. As shown in Fig. 5 treatment with Au(NHC) and Au(NHC)_2_ caused an activity reduction of about 8% and 14%, respectively. It should be underlined that in our previous proteomic study we demonstrated an increase of GAPDH amount in Au(NHC)_2_-treated cells in comparison to control, thus the percentage of activity inhibition is probably underestimated for this compound.

#### Inhibition in cell extracts

The activity of the enzyme was comparatively evaluated in the crude protein extracts of A2780 cells incubated for 5 min with increasing concentration of carbene compounds as described in Experimental Procedure. The resulting EC50 values for enzyme inhibition are reported in Table 2. All the determined EC50 values fall in the low micromolar range. Au(NHC) resulted 2.5 more effective than Au(NHC)_2_.

**Table 2:** Comparison of EC50 values of GAPDH inhibition

Compound	EC50 (μM)
Au(NHC)	2.86 ± 0.3
Au(NHC)_2_	7.13 ± 0.1

EC50 values (µM) were determined treating 10 μg of cell protein extract with aliquots of the metal complexes (in the 100 μM–0.5 μM concentration range)

### Interference of gold carbene compounds with cell migration and invasion

**Fig. 6:**
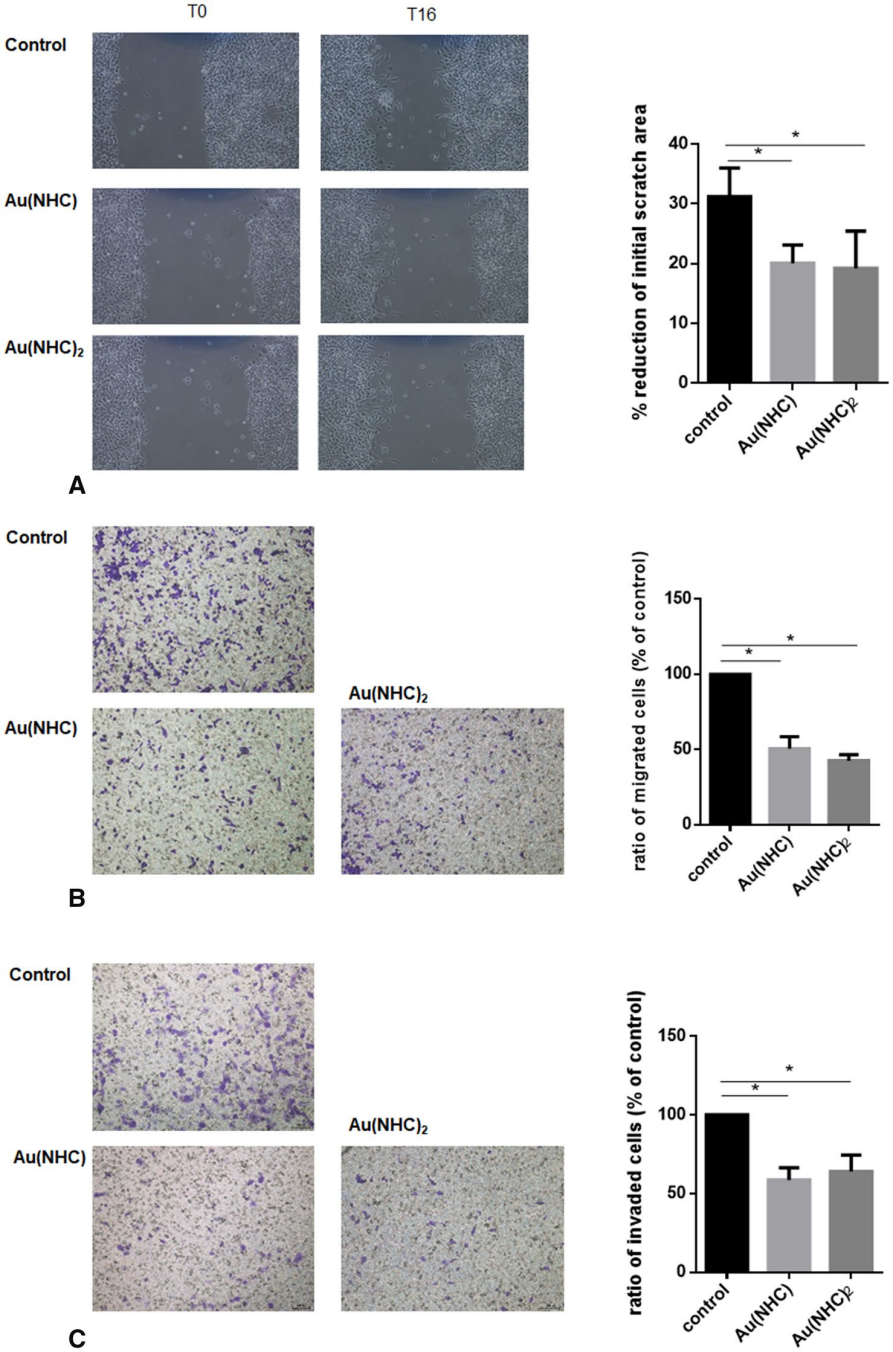
Au(NHC) and Au(NHC)_2_ inhibited migration and invasion of A2780 cells. Cell migration was determined by means of wound-healing (**A**) and trans-well assay (**B**). Cell invasion was determined on trans-well inserts pre-coated with 50 µl of Matrigel (**C**). Images are representative. The percentage of wound reduction was determined using ImageJ software. Graphs reports mean ± SD obtained in three independent experiments. The statistical analysis was carried out using one-way ANOVA test followed by Tuckey’s multiple comparisons test using GraphPad Prism v 6.0 (*p < 0.05). Image are representative

Five proteins involved in cytoskeletal organization and cell adhesion have shown an increased level of oxidation after 24 h of treatment with Au(NHC) and Au(NHC)_2_. In tubulin beta chain, actin and calponin-2 proteins, biotinylated cysteine residues have been identified by MS, and furthermore a direct interaction between actin and Au(NHC) has been demonstrated by ESI–MS experiments. All these data suggest that carbene complexes could affect cytoskeletal structure and thus, could influence cell migration and invasion that play a pivotal role in cancer progression. In order to verify this hypothesis, we investigated the ability of carbene complexes to affect these processes. Migration was evaluated by means of wound-healing and trans-well assay. As shown in Fig. 6A and B, both assays demonstrated that carbene complexes (used for 24 h at their IC50 at 72 h) attenuated migration of A2780 cells. Furthermore, they reduced invasion of A2780 cells on trans-well inserts pre-coated with Matrigel (Fig. 6C). To rule out the possibility that the inhibitory effect of Au(NHC) and Au(NHC)_2_ on migration and invasion was due to their cytotoxicity, we determined the cell viability of carbene-treated cells by means of MTT assay. Our results showed that carbene complexes (used at their IC50 at 72 h) had no significantly toxic effects on A2780 cells under the experimental conditions that involved treatment for 24 h (data not shown).

### Au(NHC)_2_ inhibits mitochondrial respiration impairing complex I function

**Fig. 7:**
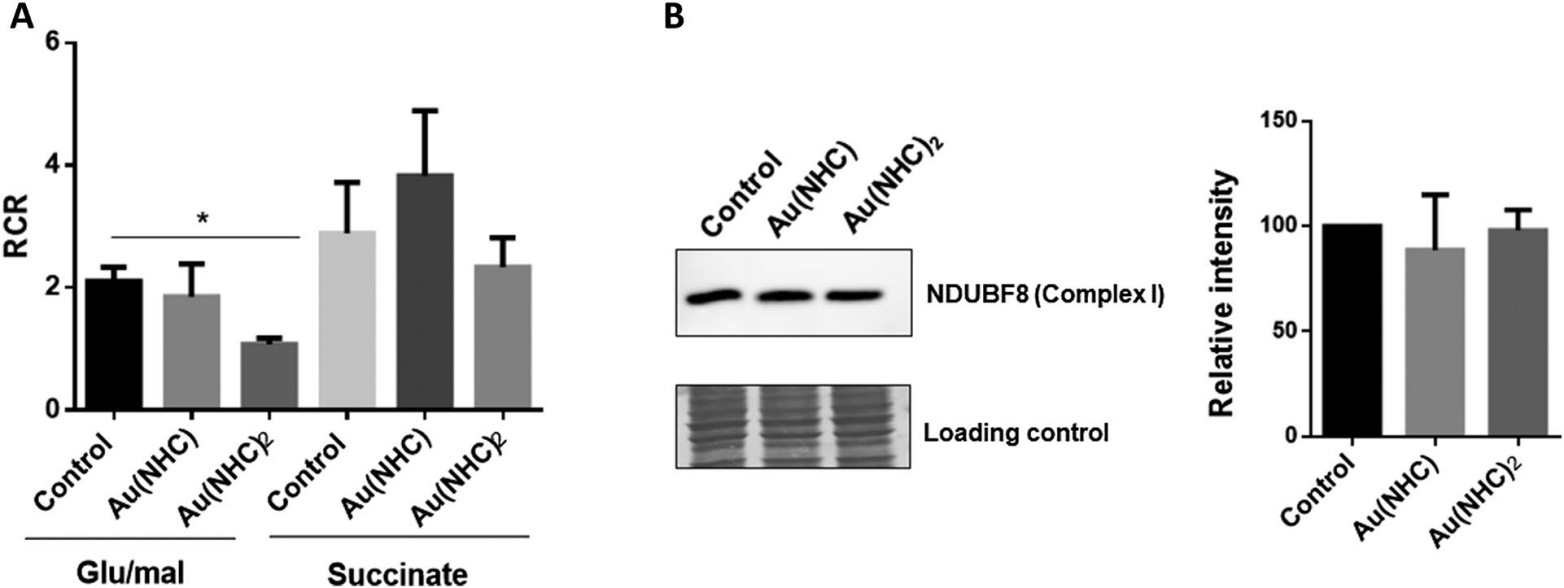
Au(NHC)_2_ affect complex I driven respiration. **A** Respiratory control ratio, RCR, was measured in A2780 permeabilized cells stimulating complex I with glutamate/malate and complex II with succinate. **B** Western blot analysis of NDUBF8 subunit of Complex I. Representative immunoblot is shown together with the matching Coomassie-stained PVDF membrane, which was used as loading control. Histogram reports the normalized mean of relative integrated density (± SD). All experiments were performed in triplicate. The statistical analysis was carried out using one-way ANOVA test followed by Tukey’s multiple comparisons test using GraphPad Prism software v 6.0 (*p < 0.05)

In our previous study, we showed that Au(NHC)_2_ causes a reduction of O_2_ consumption and an increase of ROS production together with alteration of membrane potential [16]. In the redox proteomic analysis, we failed to find mitochondrial proteins except for ATP synthase. This could be due to performing a 2D-GE on total protein extract that does not allow to efficaciously detect less abundant protein. In order to deepen the effect of gold carbene complexes on electron transport chain, we measured O_2_ consumption on A2780 cells after permeabilization with digitonin. This approach allows stimulating, with appropriate substrate, the single complexes of the electron transport chain allowing the identification of the complex mainly involved in the impairment of electron transport. Oxygen consumption was measured polarographically at 30 °C with the Clark-type oxygen electrode disc and the activity of complex II and I were separately evaluated adding to respiration medium, glutamate/malate and succinate, respectively. State 4 (mitochondrial respirations in the presence of substrate but in the absence of any added ADP and state 3 (maximal stimulated mitochondrial respirations, which is initiated by adding 1 mM ADP in the presence of substrate) were recorded. The rates of state 4 and state 3 respiration were evaluated from the linear slopes of oxygen uptake *vs* time plots. The respiratory control ratio (RCR) was calculated as the ratio of the rates of state 3 and state 4 respiration. As shown in Fig. 7A Au(NHC) does not affect complex I- and complex II-stimulated respiration, confirming our previous data. On the other hand, Au(NHC)_2_-treated cells show an impairment of complex I function whilst complex II respond normally to succinate stimulation. Furthermore, a western blot analysis was performed to evaluate the amount of NADH dehydrogenase [ubiquinone] 1 beta sub-complex subunit 8 (NDUBF8). This is a subunit of Complex I and it is considered labile when the complex is not assembled. Since no difference has been detected in the amount of this proteins between control and treated cells, the reduction of oxygen consumption caused by Au(NHC)_2_ could be ascribed to the inhibition of complex I function (Fig. 7B).

## Discussion

In the previous studies, we characterized the gold carbene compounds Au(NHC) and Au(NHC)_2_ from the chemical and biological point of view [15, 16]. In particular, we demonstrated that both compounds are able to inhibit the enzyme TrxR and to induce apoptosis in the A2780 ovarian cancer cell line but with a different efficacy, with Au(NHC)_2_ being far more active than Au(NHC). We also found that only Au(NHC)_2_ displayed a direct effect on mitochondria, causing ROS increase, alteration of the mitochondrial membrane potential and impairment of respiration.

In the present study, we have performed a redox proteomics analysis aimed at identifying proteins with cysteines that undergo oxidation upon treatment with the above-mentioned gold carbene complexes. Several studies indicated that gold compounds preferentially interact with proteins, bearing free and solvent accessible cysteines and selenocysteines. Furthermore, gold compounds were reported to generate redox state perturbation and ROS increase that, in turn, can lead to oxidation of reactive protein residues. In this context, the identification of proteins that undergo oxidation after treatment could represent a valid tool to identify potential targets that mediate the drugs’ effects.

To assess protein thiol modification, thiols can be labeled directly with biotin-tagged reagents such as biotinylated BIAM and the biotin signal can be measured by standard immunoblotting-type protocols using streptavidin-conjugated horseradish peroxidase (HRP); the loss of the biotin signal results proportional to the degree of thiol modification [20]. This technique has been extensively used both for assessing the redox state of single proteins and for redox proteomics studies [21–25].

This approach has allowed us to demonstrate that treatment with the two gold carbene complexes causes oxidation of proteins that belong to two major categories: carbohydrate metabolism, and cytoskeleton organization/cell adhesion. A few proteins of the functional classes “protein folding” and “RNA binding” are also affected. Among enzymes involved in carbohydrate metabolism, we identified: glyceraldehyde-3-phosphate dehydrogenase, alpha-enolase, fructose-bisphosphate aldolase A, fructose-bisphosphate aldolase C, phosphoglycerate kinase 1, malate dehydrogenase, cytoplasmic L-lactate dehydrogenase. All these proteins, with the exception of phosphoglycerate kinase 1, were found oxidized after Auranofin treatment in HT29 colon cancer cells indicating that gold carbene complexes behave similar to this typical and well-characterized gold(I) complex [26]. Among these enzymes, we investigated in detail the interaction between the two gold carbene complexes and GAPDH. In addition to well-known metabolic function, GAPDH displays other functions, including regulation of gene expression, intracellular membrane trafficking and cell death [27, 28]. Furthermore, GAPDH aggregation under oxidative stress has been proposed to have a pivotal role in enzyme nuclear translocation and cell death [29, 30]. The active site of human GAPDH presents two redox-sensitive amino acid residues, Cys152 and His179, which modulate the described metabolic and non-metabolic activity of GAPDH in an oxidative stress–dependent fashion. Furthermore, also the surface-exposed Cys247, mediates GAPDH multimerization and contributes to non-canonical functions of the enzyme [31, 32]. In our experiments we showed that Au(NHC) displays GAPDH inhibition in treated cells and in cell extracts and presents an elevated binding stoichiometry to GAPDH in the ESI–MS experiments suggesting that the protein metalation, somehow, leads to oxidation. Differently, Au(NHC)_2_, although showing an inhibition of the enzyme activity in treated cells comparable to Au(NHC), displayed a reduced EC50; moreover, no signal of a possible adduct was detected in the ESI–MS experiment. A possible interpretation of these results could be that the interaction between the cysteine residues and Au(NHC)_2_ is labile and transient but effective to induce cysteine oxidation. Furthermore, as already demonstrated by our group in a previous work, Au(NHC)_2_ induces a strong inhibition of thioredoxin reductase and the mitochondria functions impairment triggering an elevated ROS production and an imbalance in the redox state of the cells. Thus, within this frame, it cannot be excluded that in vivo, the observed GAPDH oxidation could be secondary to an increased ROS level and to the mitochondrial and cellular injury. [16]

Another group of oxidized proteins (calponin, actin, tubulin beta chain, F-actin-capping protein subunit alpha-1 and Serpin H1) are involved in cytoskeleton organization and cell adhesion. Actin monomers contain 5 cysteine residues: of these, only Cys 374 is fully exposed to the cytoplasm and its oxidation decreases actin polymerization [33]. In our study we found a BIAM-linked peptide containing a cysteine in position 285. Also, this cysteine is susceptible to redox regulation and its oxidation is associated with decreased ability of actin to polymerize and to interact with specific regulatory proteins including profilin [34, 35]. ESI–MS data confirmed the interaction between Au(NHC) and actin supporting the hypothesis of a direct binding. As already discussed for GAPDH, we did not detect a stable adduct between Au(NHC)_2_ and actin, thus for the latter compound we postulate an indirect mechanism of oxidation. Interestingly, Iacopetta D et al. have recently shown that two NHC-gold complexes (AuL4 and AuL7) inhibit the actin polymerization reaction and interfere with tubulin dynamic, strengthening the idea that gold carbene compounds, upon affecting cytoskeleton dynamic, could impact important pathways involved in tumor progression, such as migration and invasion [36, 37]. Several reviews have explored the role of the redox regulation of actin in these processes [38, 39]. In line with these observations, we have found that both gold carbene compounds caused a reduction of migration and invasion; thus considering the studies of Iacopetta et al., we can speculate that these compounds, by oxidizing actin and tubulin cysteine residues, might interfere with homeostasis of these proteins, impairing polymerization processes and, in turn, migration and invasion.

Finally, we have identified a few proteins involved in protein folding and RNA binding; among them Protein disulfide-isomerase A3, HSP90 alpha, HSP90 beta were also found oxidized in HT29 colon cancer treated with Auranofin [26].

Concerning mitochondria, here we confirmed that only Au(NHC)_2_ affects mitochondrial function, inhibiting complex I activity. As we already discussed in our previous study, besides the lower lipophilicity of bis(carbene) gold complex compared to the monocarbene, the bis(carbene) displayed properties like those observed in delocalized lipophilic cations (DLCs) [40], such as the ability to accumulate inside mitochondria. Targeting mitochondria has been proposed as an anticancer strategy since various cancer cells depend on OXPHOS to promote tumor aggression and response to treatment [41, 42]. Inhibitors of electron transport chain (such as metformin, tamoxifen, α-tocopheryl succinate α-TOS, 3-bromopyruvate), disrupting the function of respiratory complexes, lead to ROS increase and apoptosis. Au(NHC)_2_, inhibiting Complex I, resembles the metformin’s mode of action, although it resulted active at lower concentration (0.1 μM for Au(NHC)_2_, more than 100 μM for metformin). The inhibition of complex I could be responsible of electron leak and ROS production that, in turn, can trigger apoptosis as we have already shown in our previous paper [16]

It is also worth underlining that the effects of these gold carbene complexes, especially Au(NHC)_2_, are comparable to those of some gold (III) compounds recently investigated in our and other laboratories [43, 44]. In fact, our group recently demonstrated that three gold (III) compounds, Au_2_phen, Auoxo6 and AuL 12, inhibit the enzyme thioredoxin reductase and induce apoptotic cell death at concentration ranging from 0.1 and 4 µM. We also showed that both intrinsic and extrinsic apoptotic pathways were activated, indicating that mitochondria damage but also other not yet identified factors are involved in induction of apoptosis. Au(III) complexes, as Au(NHC)_2,_ are also able to impair energetic metabolism causing a reduction of oxygen consumption and an increase of lactate production. Overall, we can affirm that the Au(III) compounds previously studied behave, in relation to the phenomena analyzed, in a more similar way to Au(NHC)_2_, while Au(NHC) presents some differences that seem prevalently linked to its smaller effect at the mitochondrial level.

In conclusion with this study, we have deepened the understanding of the mode of action of Au(NHC) and Au(NHC)_2_ complexes, identifying a few common cellular targets and confirming their different influence on the mitochondrial function.

## Supplementary Information

Below is the link to the electronic supplementary material.

Supplementary file1 (DOCX 43 KB)

Supplementary file2 (TIF 2776 KB)

Supplementary file3 (TIF 3111 KB)
