# Supplementary material for: The effects of two gold-N-heterocyclic carbene (NHC) complexes in ovarian cancer cells: a redox proteomic study
Source: Cancer Chemother Pharmacol. 2022 May 11;89(6):809–23. doi: 10.1007/s00280-022-04438-y (PMC9135895; doi:10.1007/s00280-022-04438-y)
Supplement: Supplementary file 1 — Supplementary file1 (DOCX 43 KB) [file 280_2022_4438_MOESM1_ESM.docx]

|  |  |  |  |  |  |  |
| --- | --- | --- | --- | --- | --- | --- |
| **Spot number** | **Accession number (UniProtKB)** | **Protein name** | **Mascot score** | **N° of peptide** | **Peptide Sequence** | **Peptide with BIAM** |
| 1 | G3P_HUMAN | Glyceraldehyde-3-phosphate dehydrogenase | 485 | 7 | K.VGVNGFGR.I |  |
|  |  |  |  |  | K.AGAHLQGGAK.R |  |
|  |  |  |  |  | R.VVDLMAHMASK.E |  |
|  |  |  |  |  | R.VVDLMAHMASKE.- + Oxidation (M) |  |
|  |  |  |  |  | R.GALQNIIPASTGAAK.A |  |
|  |  |  |  |  | R.VPTANVSVVDLTCR.L |  |
|  |  |  |  |  | K.IISNASCTTNCLAPLAK.V |  |
| 2 | ENOA_HUMAN | Alpha-enolase | 1204 | 24 | K.GVPLYR.H |  |
|  |  |  |  |  | K.EGLELLK.T |  |
|  |  |  |  |  | K.YNQLLR.I |  |
|  |  |  |  |  | K.TIAPALVSK.K |  |
|  |  |  |  |  | R.IEEELGSK.A |  |
|  |  |  |  |  | K.SCNCLLLK.V |  |
|  |  |  |  |  | K.TIAPALVSKK.L |  |
|  |  |  |  |  | R.SGKYDLDFK.S |  |
|  |  |  |  |  | R.IGAEVYHNLK.N |  |
|  |  |  |  |  | K.LMIEMDGTENK.S + 2 Oxidation (M) |  |
|  |  |  |  |  | K.LNVTEQEKIDK.L |  |
|  |  |  |  |  | R.GNPTVEVDLFTSK.G |  |
|  |  |  |  |  | R.YISPDQLADLYK.S |  |
|  |  |  |  |  | K.YDLDFKSPDDPSR.Y |  |
|  |  |  |  |  | K.VVIGMDVAASEFFR.S + Oxidation (M) |  |
|  |  |  |  |  | K.VNQIGSVTESLQACK.L |  |
|  |  |  |  |  | K.IDKLMIEMDGTENK.S + Oxidation (M) |  |
|  |  |  |  |  | R.AAVPSGASTGIYEALELR.D |  |
|  |  |  |  |  | R.SGKYDLDFKSPDDPSR.Y |  |
|  |  |  |  |  | K.LAMQEFMILPVGAANFR.E + 2 Oxidation (M) |  |
|  |  |  |  |  | K.DATNVGDEGGFAPNILENK.E |  |
|  |  |  |  |  | K.FTASAGIQVVGDDLTVTNPK.R |  |
|  |  |  |  |  | K.YGKDATNVGDEGGFAPNILENK.E |  |
|  |  |  |  |  | R.SGETEDTFIADLVVGLCTGQIK.T |  |
| 3 | ENOA_HUMAN | Alpha-enolase | 201 | 4 | K.VVIGMDVAASEFFR.S + Oxidation (M) |  |
|  |  |  |  |  | K.VNQIGSVTESLQACK.L |  |
|  |  |  |  |  | R.AAVPSGASTGIYEALELR.D |  |
|  |  |  |  |  | K.FTASAGIQVVGDDLTVTNPK.R |  |
| 4 | PCBP1_HUMAN | Poly(rC)-binding protein 1 | 466 | 11 | K.EVGSIIGK.K | GGCKIKEIR |
|  |  |  |  |  | R.QMSGAQIK.I + Oxidation (M) |  |
|  |  |  |  |  | R.IREESGAR.I |  |
|  |  |  |  |  | R.QGANINEIR.Q |  |
|  |  |  |  |  | K.IANPVEGSSGR.Q |  |
|  |  |  |  |  | R.INISEGNCPER.I |  |
|  |  |  |  |  | R.IITLTGPTNAIFK.A |  |
|  |  |  |  |  | R.LVVPATQCGSLIGK.G |  |
|  |  |  |  |  | R.AITIAGVPQSVTECVK.Q |  |
|  |  |  |  |  | R.ESTGAQVQVAGDMLPNSTER.A + Oxidation (M) |  |
| 5 | ENOA_HUMAN | Alpha-enolase | 68 | 2 | K.VNQIGSVTESLQACK.L |  |
|  |  |  |  |  | K.FTASAGIQVVGDDLTVTNPK.R |  |
| 6 | ALDOA_HUMAN | Fructose-bisphosphate aldolase A | 1070 | 21 | K.GGVVGIK.V |  |
|  |  |  |  |  | K.DGADFAK.W |  |
|  |  |  |  |  | K.VLAAVYK.A |  |
|  |  |  |  |  | R.ALQASALK.A |  |
|  |  |  |  |  | K.ELSDIAHR.I |  |
|  |  |  |  |  | K.GGVVGIKVDK.G |  |
|  |  |  |  |  | R.QLLLTADDR.V |  |
|  |  |  |  |  | K.KELSDIAHR.I |  |
|  |  |  |  |  | R.ALANSLACQGK.Y |  |
|  |  |  |  |  | K.SKGGVVGIKVDK.G |  |
|  |  |  |  |  | K.RALANSLACQGK.Y |  |
|  |  |  |  |  | K.GILAADESTGSIAK.R |  |
|  |  |  |  |  | K.ADDGRPFPQVIK.S |  |
|  |  |  |  |  | M.PYQYPALTPEQK.K |  |
|  |  |  |  |  | K.GILAADESTGSIAKR.L |  |
|  |  |  |  |  | R.LQSIGTENTEENR.R |  |
|  |  |  |  |  | M.PYQYPALTPEQKK.E |  |
|  |  |  |  |  | R.LQSIGTENTEENRR.F |  |
|  |  |  |  |  | K.RLQSIGTENTEENRR.F |  |
| 6 | ALDOC_HUMAN | Fructose-bisphosphate aldolase C | 243 | 4 | K.DGADFAK.W | CIGGVIFFHETLYQKDDNGVPFVR |
|  |  |  |  |  | K.VLAAVYK.A |  |
|  |  |  |  |  | K.GVVPLAGTDGETTTQGLDGLSER.C |  |
|  |  |  |  |  | K.VDKGVVPLAGTDGETTTQGLDGLSER.C |  |
| 6 | PGK1_HUMAN | Phosphoglycerate kinase 1 | 191 | 5 | R.VDFNVPMK.N + Oxidation (M) | ACANPAAGSVILLENLRFHVEEEGKGK |
|  |  |  |  |  | R.FHVEEEGKGK.D |  |
|  |  |  |  |  | R.AHSSMVGVNLPQK.A + Oxidation (M) |  |
|  |  |  |  |  | K.LGDVYVNDAFGTAHR.A |  |
|  |  |  |  |  | R.GCITIIGGGDTATCCAK.W |  |
| 7 | MDHC_HUMAN | Malate dehydrogenase, cytoplasmic | 276 | 6 | K.LGVTANDVK.N | AICDHVR |
|  |  |  |  |  | R.KLSSAMSAAK.A + Oxidation (M) |  |
|  |  |  |  |  | K.ENFSCLTR.L |  |
|  |  |  |  |  | K.SQGAALDKYAK.K |  |
|  |  |  |  |  | K.GEFVTTVQQR.G |  |
|  |  |  |  |  | K.DVIATDKEDVAFK.D |  |
| 7 | CNN2_HUMAN | Calponin-2 | 471 | 9 | K.YSEKQER.N | CASQSGMTAYGTR |
|  |  |  |  |  | K.GLQSGVDIGVK.Y |  |
|  |  |  |  |  | K.GPSYGLSAEVK.N |  |
|  |  |  |  |  | K.DGTILCTLMNK.L + Oxidation (M) |  |
|  |  |  |  |  | K.TKGLQSGVDIGVK.Y |  |
|  |  |  |  |  | K.CASQVGMTAPGTR.R |  |
|  |  |  |  |  | K.CASQSGMTAYGTR.R |  |
|  |  |  |  |  | K.AGQCVIGLQMGTNK.C |  |
|  |  |  |  |  | K.CASQVGMTAPGTRR.H |  |
| 8 | LDHB_HUMAN | L-lactate dehydrogenase B chain  [Homo sapiens] | 102 | 2 | K.IVVVTAGVR.Q |  |
|  |  |  |  |  | R.VIGSGCNLDSAR.F |  |
| 9 | F10A1_HUMAN | Hsc70-interacting protein  [Homo sapiens] | 359 | 6 | R.LAILYAK.R | AFVKMCK |
|  |  |  |  |  | R.EWVESMGGK.V |  |
|  |  |  |  |  | K.AIDLFTDAIK.L |  |
|  |  |  |  |  | K.LDYDEDASAMLK.E +  Oxidation (M) |  |
|  |  |  |  |  | R.AIEINPDSAQPYK.W |  |
|  |  |  |  |  | K.VAAIEALNDGELQK.A |  |
| 10 | GLRX3_HUMAN | Glutaredoxin-3 [Homo sapiens] | 347 | 5 | K.ELPQVSFVK.L |  |
|  |  |  |  |  | K.GELVGGLDIVK.E |  |
|  |  |  |  |  | K.ENGELLPILR.G |  |
|  |  |  |  |  | K.LEAEGVPEVSEK.Y |  |
|  |  |  |  |  | K.ELEASEELDTICPK.A |  |
| 11 | ACTB_HUMAN | Actin, cytoplasmic 1  [Homo sapiens] | 186 | 2 | K.AGFAGDDAPR.A | CDVDIRK |
|  |  |  |  |  | K.SYELPDGQVITIGNER.F |  |
| 12 | TBB5_HUMAN | beta-tubulin [Homo sapiens] | 231 | 6 | K.TAVCDIPPR.G | MREIVHIQAGQCGNQIGAK |
|  |  |  |  |  | R.FPGQLNADLR.K | NMMAACDPRHGRYLTVAAVFR |
|  |  |  |  |  | K.LAVNMVPFPR.L |  |
|  |  |  |  |  | R.ISVYYNEATGGK.Y |  |
|  |  |  |  |  | K.EVDEQMLNVQNK.N |  |
|  |  |  |  |  | R.AILVDLEPGTMDSVR.S + Oxidation (M) |  |
| 13 | CAZA1_HUMAN | F-actin-capping protein subunit alpha-1  [Homo sapiens] | 86 | 2 | R.LLLNNDNLLR.E | - |
|  |  |  |  |  | K.EASDPQPEEADGGLK.S |  |
| 14 | SERA_HUMAN | D-3-phosphoglycerate dehydrogenase | 641 | 12 | R.VVNCAR.G | VVNCAR |
|  |  |  |  |  | K.TLGILGLGR.I |  |
|  |  |  |  |  | K.QADVNLVNAK.L |  |
|  |  |  |  |  | R.GGIVDEGALLR.A |  |
|  |  |  |  |  | K.VTADVINAAEK.L |  |
|  |  |  |  |  | K.ILQDGGLQVVEK.Q |  |
|  |  |  |  |  | K.GTIQVITQGTSLK.N |  |
|  |  |  |  |  | K.VLISDSLDPCCR.K |  |
|  |  |  |  |  | R.AGTGVDNVDLEAATR.K |  |
|  |  |  |  |  | R.AGTGVDNVDLEAATRK.G |  |
|  |  |  |  |  | R.CGEEIAVQFVDMVK.G +  Oxidation (M) |  |
|  |  |  |  |  | R.TQTSDPAMLPTMIGLLAEAGVR.L +  2 Oxidation (M) | |
| 15 | SERPH_HUMAN | Serpin H1 | 240 | 5 | K.AVAISLPK.G | - |
|  |  |  |  |  | K.AVLSAEQLR.D |  |
|  |  |  |  |  | K.GVVEVTHDLQK.H |  |
|  |  |  |  |  | K.LQIVEMPLAHK.L + Oxidation (M) |  |
|  |  |  |  |  | K.HLAGLGLTEAIDKNK.A |  |
| 16 | PDIA3_HUMAN | Protein disulfide-isomerase | 1277 | 27 | K.GIVPLAK.V |  |
|  |  |  |  |  | K.DPNIVIAK.M |  |
|  |  |  |  |  | K.LNFAVASR.K |  |
|  |  |  |  |  | R.LKGIVPLAK.V |  |
|  |  |  |  |  | R.TADGIVSHLK.K |  |
|  |  |  |  |  | K.YGVSGYPTLK.I |  |
|  |  |  |  |  | R.TADGIVSHLKK.Q |  |
|  |  |  |  |  | K.FVMQEEFSR.D +  Oxidation (M) |  |
|  |  |  |  |  | R.LAPEYEAAATR.L |  |
|  |  |  |  |  | K.LSKDPNIVIAK.M |  |
|  |  |  |  |  | R.DGEEAGAYDGPR.T |  |
|  |  |  |  |  | R.GFPTIYFSPANK.K |  |
|  |  |  |  |  | K.RLAPEYEAAATR.L |  |
|  |  |  |  |  | R.FLQDYFDGNLK.R |  |
|  |  |  |  |  | K.SEPIPESNDGPVK.V |  |
|  |  |  |  |  | R.ELSDFISYLQR.E |  |
|  |  |  |  |  | K.VDCTANTNTCNK.Y |  |
|  |  |  |  |  | K.FEDKTVAYTEQK.M |  |
|  |  |  |  |  | R.GFPTIYFSPANKK.L |  |
|  |  |  |  |  | K.FVMQEEFSRDGK.A +  Oxidation (M) |  |
|  |  |  |  |  | R.FLQDYFDGNLKR.Y |  |
|  |  |  |  |  | R.EATNPPVIQEEKPK.K |  |
|  |  |  |  |  | K.IFRDGEEAGAYDGPR.T |  |
|  |  |  |  |  | K.MDATANDVPSPYEVR.G +  Oxidation (M) |  |
|  |  |  |  |  | K.QAGPASVPLRTEEEFK.K +  Gln->pyro-Glu (N-term Q) | |
|  |  |  |  |  | R.YLKSEPIPESNDGPVK.V |  |
|  |  |  |  |  | K.VDCTANTNTCNKYGVSGYPTLK.I |  |
| 17 | PDIA3_HUMAN | Protein disulfide-isomerase A3 | 434 | 11 | K.GIVPLAK.V |  |
|  |  |  |  |  | K.LNFAVASR.K |  |
|  |  |  |  |  | K.YGVSGYPTLK.I |  |
|  |  |  |  |  | K.FVMQEEFSR.D + Oxidation (M) |  |
|  |  |  |  |  | R.LAPEYEAAATR.L |  |
|  |  |  |  |  | K.LSKDPNIVIAK.M |  |
|  |  |  |  |  | R.DGEEAGAYDGPR.T |  |
|  |  |  |  |  | R.GFPTIYFSPANK.K |  |
|  |  |  |  |  | K.SEPIPESNDGPVK.V |  |
|  |  |  |  |  | K.IFRDGEEAGAYDGPR.T |  |
|  |  |  |  |  | K.MDATANDVPSPYEVR.G +  Oxidation (M) |  |
| 18 | TCPE_HUMAN | T-complex protein 1 subunit epsilon | 552 | 12 | R.AVTIFIR.G |  |
|  |  |  |  |  | K.MIIEEAK.R |  |
|  |  |  |  |  | K.LMVELSK.S + Oxidation (M) |  |
|  |  |  |  |  | R.FSELTAEK.L |  |
|  |  |  |  |  | R.TSLGPNGLDK.M |  |
|  |  |  |  |  | K.MLVIEQCK.N |  |
|  |  |  |  |  | R.IADGYEQAAR.V |  |
|  |  |  |  |  | K.DTEPLIQTAK.T |  |
|  |  |  |  |  | K.LDVTSVEDYK.A |  |
|  |  |  |  |  | K.FEEMIQQIK.E + Oxidation (M) |  |
|  |  |  |  |  | K.DKMLVIEQCK.N + Oxidation (M) |  |
|  |  |  |  |  | K.QQISLATQMVR.M + Oxidation (M) |  |
| 18 | TCPQ_HUMAN | T-complex protein 1 subunit theta | 405 | 10 | K.YNIMLVR.L + Oxidation (M) |  |
|  |  |  |  |  | K.ETEGDVTSVK.D |  |
|  |  |  |  |  | R.AVDDGVNTFK.V |  |
|  |  |  |  |  | K.LATNAAVTVLR.V |  |
|  |  |  |  |  | R.DIDEVSSLLR.T |  |
|  |  |  |  |  | K.FAEAFEAIPR.A |  |
|  |  |  |  |  | K.TAEELMNFSK.G + Oxidation (M) |  |
|  |  |  |  |  | K.GEENLMDAQVK.A +  Oxidation (M) |  |
|  |  |  |  |  | K.AIADTGANVVVTGGK.V |  |
|  |  |  |  |  | R.GSTDNLMDDIER.A +  Oxidation (M) |  |
| 19 | HS90B_HUMAN | Heat shock protein HSP 90-beta | 310 | 5 | K.SIYYITGESK.E | AKFENLCK |
|  |  |  |  |  | K.ADLINNLGTIAK.S | KCLELFSELAEDKENYK |
|  |  |  |  |  | R.ELISNASDALDK.I | CLELFSELAEDKENYKK |
|  |  |  |  |  | R.TLTLVDTGIGMTK.A + Oxidation (M) |  |
|  |  |  |  |  | R.GVVDSEDLPLNISR.E |  |
| 19 | HS90A_HUMAN | Heat shock protein HSP 90-alpha | 290 | 5 | K.FYEQFSK.N | DYCTR |
|  |  |  |  |  | K.ADLINNLGTIAK.S | DYCTRMKENQK |
|  |  |  |  |  | R.TLTIVDTGIGMTK.A + Oxidation (M) |  |
|  |  |  |  |  | R.GVVDSEDLPLNISR.E |  |
|  |  |  |  |  | R.NPDDITNEEYGEFYK.S |  |
| 20 | TCPE_HUMAN | T-complex protein 1 subunit epsilon | 1098 | 24 | K.LMVELSK.S |  |
|  |  |  |  |  | R.AVTIFIR.G |  |
|  |  |  |  |  | K.MIIEEAK.R |  |
|  |  |  |  |  | R.LMGLEALK.S +  Oxidation (M) |  |
|  |  |  |  |  | R.FSELTAEK.L |  |
|  |  |  |  |  | K.MIIEEAKR.S |  |
|  |  |  |  |  | R.TSLGPNGLDK.M |  |
|  |  |  |  |  | K.MLVIEQCK.N +  Oxidation (M) |  |
|  |  |  |  |  | R.IADGYEQAAR.V |  |
|  |  |  |  |  | K.SRLMGLEALK.S +  Oxidation (M) |  |
|  |  |  |  |  | K.FEEMIQQIK.E |  |
|  |  |  |  |  | K.LDVTSVEDYK.A |  |
|  |  |  |  |  | R.SLHDALCVIR.N |  |
|  |  |  |  |  | K.QQHVIETLIGK.K +  Gln->pyro-Glu (N-term Q) |  |
|  |  |  |  |  | K.QQISLATQMVR.M |  |
|  |  |  |  |  | R.DVDFELIKVEGK.V |  |
|  |  |  |  |  | K.EKFEEMIQQIK.E +  Oxidation (M) |  |
|  |  |  |  |  | R.RDVDFELIKVEGK.V |  |
|  |  |  |  |  | K.IAILTCPFEPPKPK.T |  |
|  |  |  |  |  | K.LGFAGLVQEISFGTTK.D |  |
|  |  |  |  |  | K.GVIVDKDFSHPQMPK.K |  |
|  |  |  |  |  | R.WVGGPEIELIAIATGGR.I |  |
|  |  |  |  |  | R.VVYGGGAAEISCALAVSQEADK.C |  |
|  |  |  |  |  | K.MMVDKDGDVTVTNDGATILSMMDVDHQIAK.L +  3 Oxidation (M) | |
| 21 | PCBP2_HUMAN | Poly(rC)-binding protein 2 | 142 | 4 | K.IANPVEGSTDR.Q | GGCKIKEIR |
|  |  |  |  |  | R.INISEGNCPER.I |  |
|  |  |  |  |  | R.LVVPASQCGSLIGK.G |  |
|  |  |  |  |  | R.ESTGAQVQVAGDMLPNSTER.A +  Oxidation (M) | |
| 22 | RBM4_HUMAN | RNA-binding protein 4 | 275 | 7 | K.LFIGNLPR.E | SLFEQYGKVLECDIIK |
|  |  |  |  |  | R.NSLYDMAR.Y |  |
|  |  |  |  |  | R.GLDNTEFQGKR.M |  |
|  |  |  |  |  | R.TAPGMGDQSGCYR.C |  |
|  |  |  |  |  | K.LHVGNISPTCTNK.E |  |
|  |  |  |  |  | R.LRTAPGMGDQSGCYR.C + Oxidation (M) |  |
|  |  |  |  |  | R.VADLTEQYNEQYGAVR.T |  |
| 22 | BUB3_HUMAN | Mitotic checkpoint protein BUB3 | 151 | 3 | K.VYTLSVSGDR.L | YQTRCIR |
|  |  |  |  |  | R.TPCNAGTFSQPEK.V |  |
|  |  |  |  |  | R.VAVEYLDPSPEVQK.K |  |
| 23 | PEF1_HUMAN | Peflin | 50 | 2 | K.NLFQQYDR.D |  |
|  |  |  |  |  | R.SANPAMQLDR.F |  |
| 24 | B3AT_HUMAN | Band 3 anion transport protein | 241 | 5 | K.VYVELQELVMDEK.N |  |
|  |  |  |  |  | R.LQEAAELEAVELPVPIR.F |  |
|  |  |  |  |  | K.ATFDEEEGRDEYDEVAMPV.- |  |
|  |  |  |  |  | K.GTVLLDLQETSLAGVANQLLDR.F |  |
|  |  |  |  |  | R.VIGDFGVPISILIMVLVDFFIQDTYTQK.L |  |
| 25 | IMDH2_HUMAN | Inosine-5'-monophosphate dehydrogenase 2 | 355 | 6 | R.LVGIISSR.D | HGFCGIPITDTGR |
|  |  |  |  |  | K.EANEILQR.S |  |
|  |  |  |  |  | R.AMMYSGELK.F |  |
|  |  |  |  |  | K.NLIDAGVDALR.V |  |
|  |  |  |  |  | K.VAQGVSGAVQDK.G |  |
|  |  |  |  |  | R.EDLVVAPAGITLK.E |  |
| 26 | ATPA_HUMAN | ATP synthase subunit alpha, mitochondrial | 559 | 12 | K.APGIIPR.I |  |
|  |  |  |  |  | R.ELIIGDR.Q |  |
|  |  |  |  |  | R.STVAQLVK.R |  |
|  |  |  |  |  | R.QMSLLLR.R + Oxidation (M) |  |
|  |  |  |  |  | K.LELAQYR.E |  |
|  |  |  |  |  | K.AVDSLVPIGR.G |  |
|  |  |  |  |  | R.VVDALGNAIDGK.G |  |
|  |  |  |  |  | K.TSIAIDTIINQK.R |  |
|  |  |  |  |  | K.TGTAEMSSILEER.I + Oxidation (M) |  |
|  |  |  |  |  | R.ILGADTSVDLEETGR.V |  |
|  |  |  |  |  | R.TGAIVDVPVGEELLGR.V |  |
|  |  |  |  |  | R.VVDALGNAIDGKGPIGSK.T |  |
| 26 | GSHR_HUMAN | Glutathione reductase, mitochondrial | 227 | 3 | K.GIYAVGDVCGK.A |  |
|  |  |  |  |  | K.ALLTPVAIAAGR.K |  |
|  |  |  |  |  | R.LNAIYQNNLTK.S |  |
