# Supplementary figures and images for: The effects of two gold-N-heterocyclic carbene (NHC) complexes in ovarian cancer cells: a redox proteomic study
Source: Cancer Chemother Pharmacol. 2022 May 11;89(6):809–23. doi: 10.1007/s00280-022-04438-y (PMC9135895; doi:10.1007/s00280-022-04438-y)

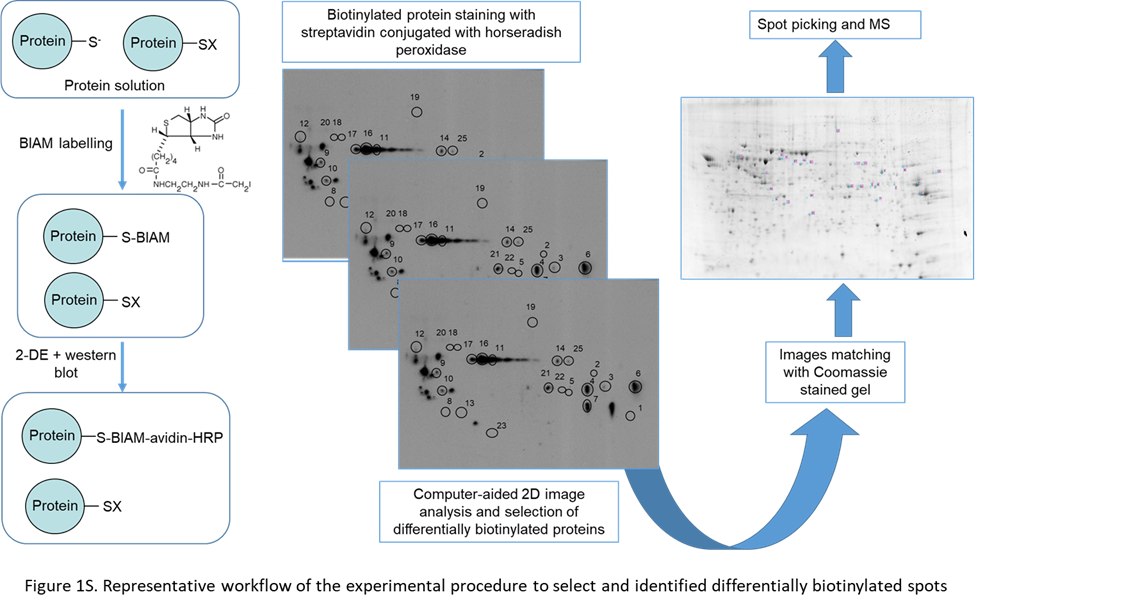

Supplement: Supplementary file 2 — Supplementary file2 (TIF 2776 KB) [file 280_2022_4438_MOESM2_ESM.tif]

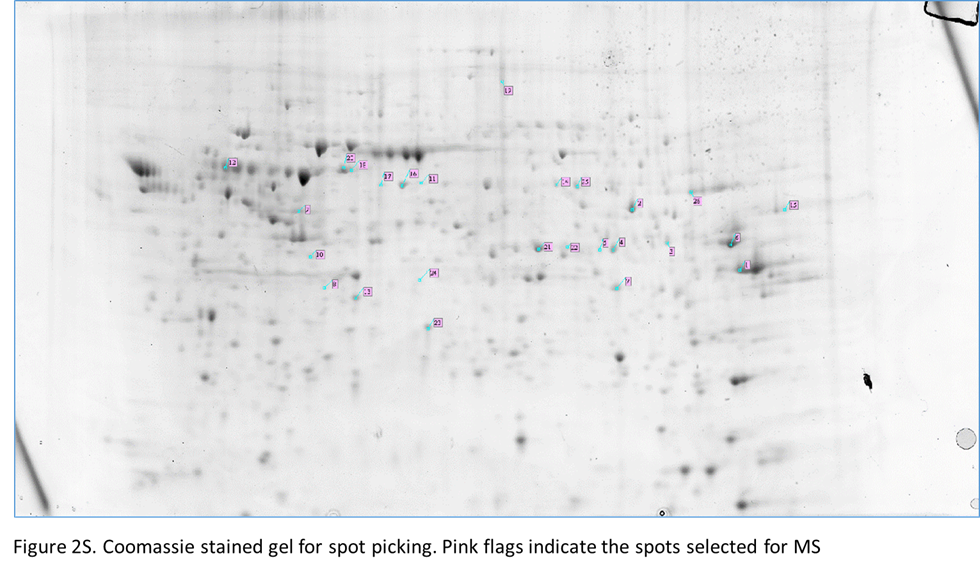

Supplement: Supplementary file 3 — Supplementary file3 (TIF 3111 KB) [file 280_2022_4438_MOESM3_ESM.tif]
